# Correlating the nanostructure of Al-oxide with deposition conditions and dielectric contributions of two-level systems in perspective of superconducting quantum circuits

**DOI:** 10.1038/s41598-018-26066-4

**Published:** 2018-05-21

**Authors:** S. Fritz, A. Seiler, L. Radtke, R. Schneider, M. Weides, G. Weiß, D. Gerthsen

**Affiliations:** 1 Laboratory for Electron Microscopy, Karlsruhe Institute of Technology (KIT), 76131 Karlsruhe, Germany; 2 Physikalisches Institut, Karlsruhe Institute of Technology (KIT), 76131 Karlsruhe, Germany; 3 School of Engineering, University of Glasgow, G12 8QQ Glasgow, United Kingdom

## Abstract

This work is concerned with Al/Al-oxide(AlO_x_)/Al-layer systems which are important for Josephson-junction-based superconducting devices such as quantum bits. The device performance is limited by noise, which has been to a large degree assigned to the presence and properties of two-level tunneling systems in the amorphous AlO_x_ tunnel barrier. The study is focused on the correlation of the fabrication conditions, nanostructural and nanochemical properties and the occurrence of two-level tunneling systems with particular emphasis on the AlO_x_-layer. Electron-beam evaporation with two different processes and sputter deposition were used for structure fabrication, and the effect of illumination by ultraviolet light during Al-oxide formation is elucidated. Characterization was performed by analytical transmission electron microscopy and low-temperature dielectric measurements. We show that the fabrication conditions have a strong impact on the nanostructural and nanochemical properties of the layer systems and the properties of two-level tunneling systems. Based on the understanding of the observed structural characteristics, routes are suggested towards the fabrication of Al/AlO_x_/Al-layers systems with improved properties.

## Introduction

Intense efforts have been devoted to possible realizations of quantum information processing during the past two decades. One of the technologically most advanced approaches at present relies on superconducting circuits^1^. Their basic units, quantum bits (qubits), contain Josephson junctions (JJs) which are worldwide mainly fabricated on the basis of Al/AlO_x_/Al-layer systems. Complex superconducting circuits will contain a large number of qubits and JJs whose properties, in particular coherence and relaxation times, and homogeneity will be crucial for further progress of the technology. Coherence times up to 10^−3^ s have been achieved^2^ by the elimination of various sources of noise in well-shielded single-qubit experiments, but further improvements are necessary for complex quantum circuits. This has led to intense investigations of sources of noise, such as flux noise^3^, fluctuations of the critical current^4^, charge noise induced by surface and gap states^5^, and contaminations at interfaces^6^. A major source of noise and decoherence in qubits is assigned to two-level defects, commonly denoted as two-level systems (TLS). Several theoretical and some experimental studies on TLS properties resulted in suggestions regarding possible sources of TLS^6–10^ but, despite of these efforts, the microscopic origin of TLS is still under debate.

The properties of individual TLS were investigated in only few experimental studies. Using qubits, resonance techniques were applied to spectroscopically identify TLS in the microwave frequency regime (few GHz) which are characterized by electric and elastic dipole moments^7,8^. Recent theoretical studies focused on the intrinsic properties of the AlO_x_-tunnel barrier, where bistable defects, e.g. delocalized oxygen atoms, could be a source of TLS^9,10^. It has been indeed well known for decades that two-level atomic tunneling systems dominate the low-temperature properties of glasses and other disordered solids^11,12^. Small groups of atoms, the nature of which is not further specified, are assumed to tunnel between two energetically almost equivalent configurations. The TLS are modeled as particles in double-well potentials with a broad distribution of the relevant parameters. The resulting density of states is essentially constant, though material-specific, causing a logarithmic dependence of the dielectric permittivity as a function of temperature^13,14^. For the case of a disordered AlO_x_-film, this logarithmic dependence scales with the combined effect of TLS density of states per volume and energy and the squared average TLS dipole moment^15^.

Numerous studies are concerned with the fabrication and properties of AlO_x_-based tunnel barriers. The standard procedure involves thermal or plasma-assisted surface oxidation of an Al-layer^16–20^, typically performed at room temperature. This is a self-limiting process and results in an amorphous AlO_x_-layer with a thickness of 1–3 nm. Optimization of the tunnel barrier can be achieved by studying, e.g., the critical current density of JJs as a function of oxidation pressure and oxidation time^21^. A variety of different techniques were used in the past to study JJ properties, but only few dedicated analytical transmission electron microscopy (TEM) investigations were undertaken to analyze the structural and chemical properties of amorphous AlO_x_-tunnel barriers on the nanoscale^22–25^. A recent study by Zeng *et al*.^26^ focuses on the analysis of locally acquired pair distribution functions and concludes that the AlO_x_-tunnel barrier is O-deficient. These results may give hints on the microscopic nature of TLS. We note that other applications of Al/AlO_x_/Al- and Nb/AlO_x_/Nb-based JJs such as X-ray detectors^27^, voltage standards^28^ and superconducting quantum devices^29^ will also profit from optimized JJs because their properties also sensitively depend on noise.

To make further progress towards the reduction of noise induced by TLS, in this work the nanochemical and nanostructural properties of Al/AlO_x_/Al-layer systems are correlated with deposition conditions and low-temperature capacitance measurements to assess the TLS contribution to the dielectric permittivity. TEM and scanning transmission electron microscopy (STEM) combined with electron energy loss spectroscopy (EELS) were applied to analyze the nanochemistry and nanostructure of four differently fabricated Al/AlO_x_/Al-layer systems. AlO_x_-layers with a thickness of 20 to 30 nm are necessary to obtain a sufficiently high electric resistance and small electric field strength for capacitance measurements. We find pronounced differences of the dielectric TLS contribution and structural and chemical properties of the analyzed Al/AlO_x_/Al-layer systems, which suggest measures on how to improve the properties of the AlO_x_-tunnel barriers in JJs in the future.

## Experimental Results

Four differently produced Al/AlO_x_/Al-layer systems were investigated. The first sample was fabricated in a *MEB 550S (PLASSYS Bestek, Marolles-en-Hurepoix, FR)* electron-beam deposition system. After deposition of an Al-layer, dynamic oxidation with pure O_2_ at room temperature was applied to form the AlO_x_-layer. The same system was used to fabricate a second sample, where AlO_x_-formation took place under UV-illumination to enhance dynamic oxidation. These two samples are denoted as EBPlas and EBPlas-UV. AlO_x_-layer thicknesses between 20 and 30 nm were obtained by repeating Al-deposition and dynamic oxidation several times to achieve a sufficiently high electric resistance for the capacitance measurements (cf. Methods). The third sample, denoted as EBLes, was fabricated in a different electron-beam deposition system (*PVD 75, Kurt J. Lesker Company, Hastings, UK*). Electron-beam deposition was applied not only for the Al-layers but also for the AlO_x_-layer using stoichiometric Al_2_O_3_-pellets. The fourth sample SPUT was fabricated in a home-built sputter deposition system. Al-deposition was performed using an Al-target in an Ar-plasma, while the AlO_x_-film was deposited from the same Al-target in a reactive Ar/O-plasma.

**Figure 1:**
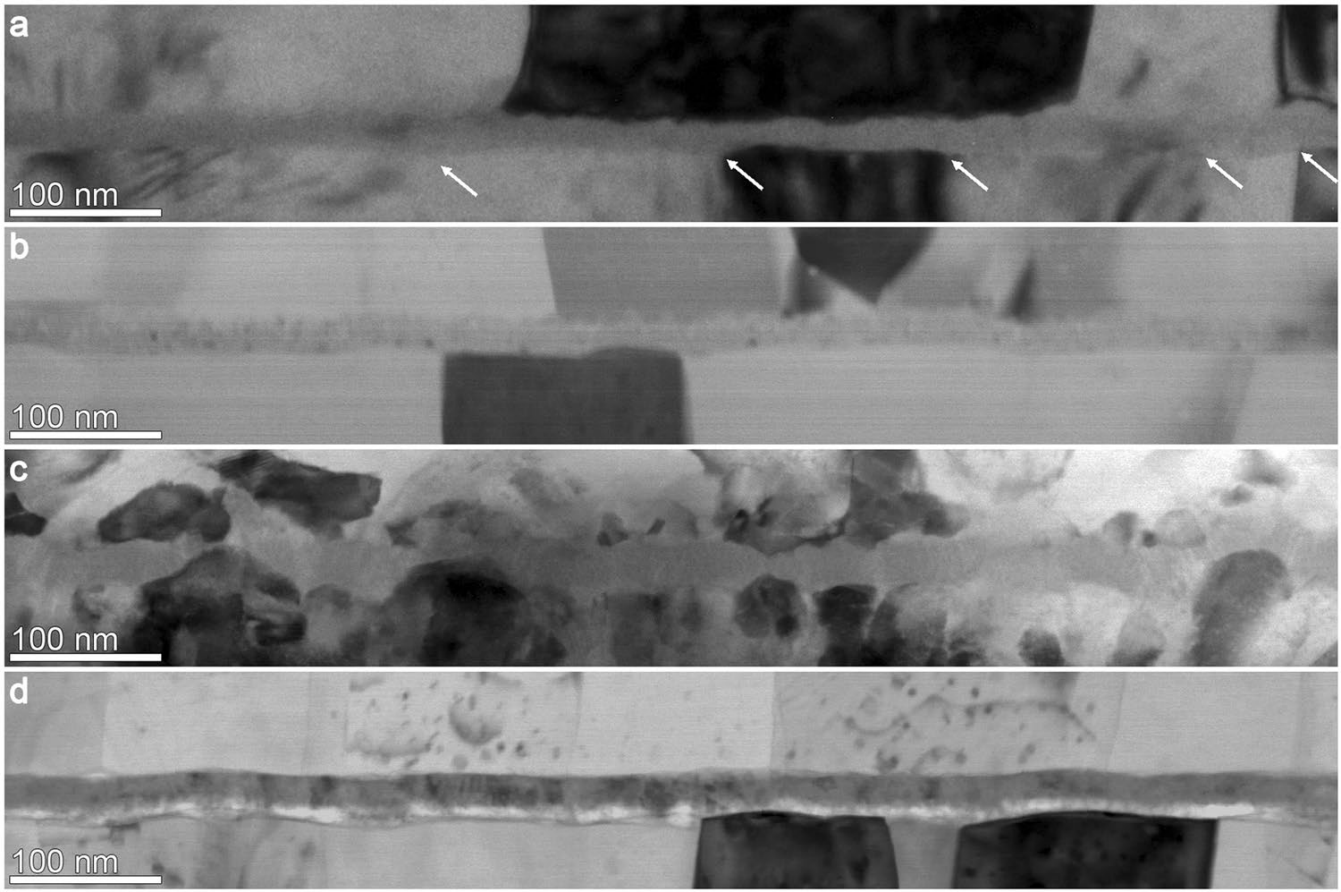
Overview bright-field TEM cross-section images of (**a**) EBPlas, (**b**) EBPlas-UV (**c**) EBLes and (**d**) SPUT. Grain boundaries in the bottom Al-layer of EBPlas (a) are marked by white arrows.

Figure 1 shows overview bright-field (BF) TEM cross-section images of all samples. Grains with a large average lateral size of (196 ± 89) nm are observed in the lower Al-layer of EBPlas (Fig. 1a). The rather homogenous layer thickness leads to a small corrugation of the lower Al/AlO_x_-interface. The thickness of the AlO_x_-layer varies considerably and increases the corrugation at the upper Al/AlO_x_-interface. The most pronounced thickness variations and distortions of the AlO_x_-layer are found in regions where Al-grain boundaries intersect the Al/AlO_x_-interface (white arrows in Fig. 1a). Thickness homogeneity of the AlO_x_-layer and interface corrugation are improved in EBPlas-UV (Fig. 1b). The average lateral grain size (189 ± 90) nm in the lower Al-layer is similar as in EBPlas. The microstructure of EBLes (Fig. 1c) differs considerably from EBPlas and EBPlas-UV although it was also fabricated in an electron-beam deposition system. The Al-layers are substantially corrugated and consist of small Al-grains with sizes of (54 ± 23) nm. The AlO_x_-layer varies strongly in thickness.

The forth sample SPUT (Fig. 1d) contains Al-layers with an average lateral grain size of (117 ± 62) nm and an AlO_x_-layer with the most homogeneous thickness of all samples. However, the AlO_x_-layer appears to be subdivided into a lower sublayer with bright contrast and a darker upper part.

**Figure 2:**
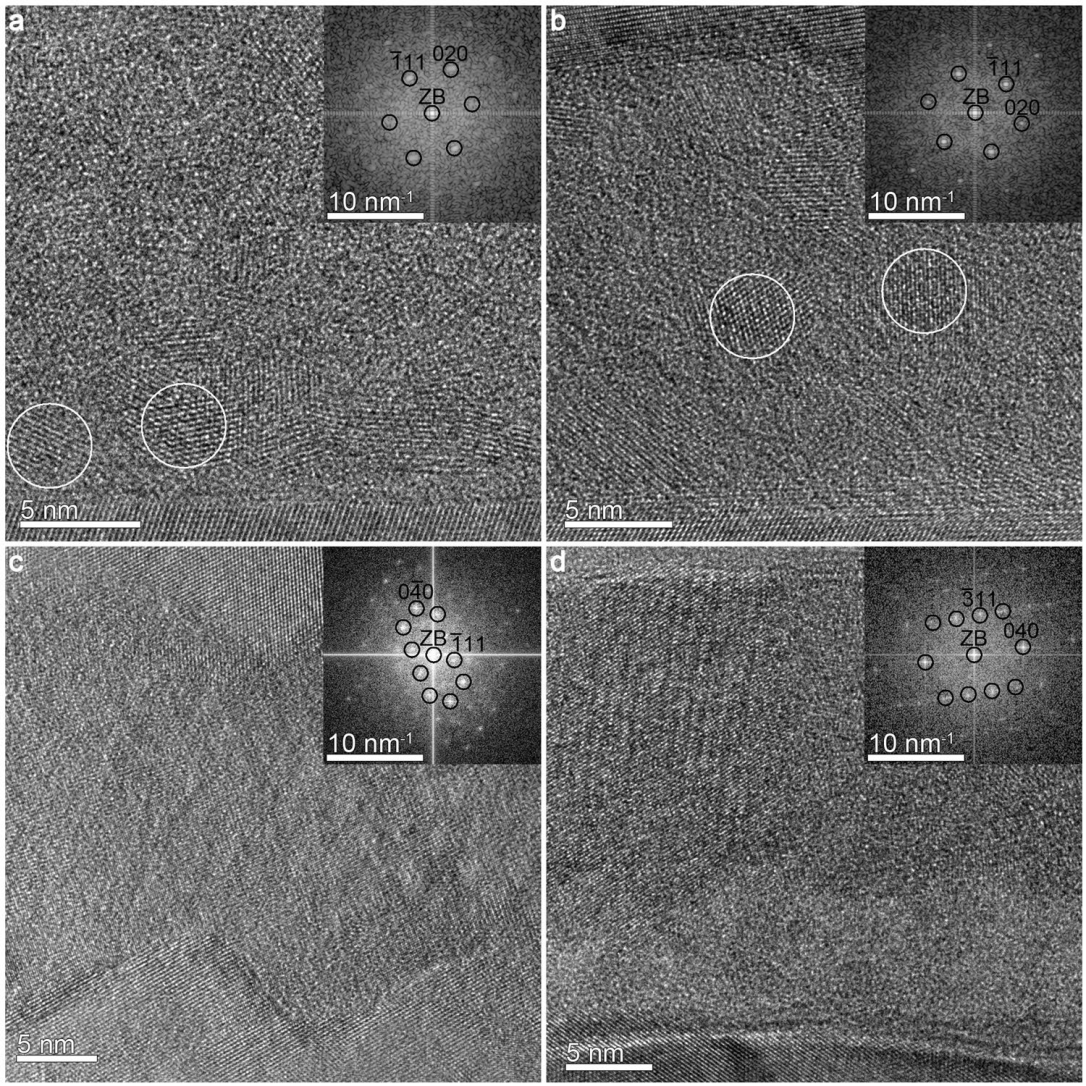
High-resolution cross-section TEM images of (**a**) EBPlas with FT pattern of an Al-nanocrystal in [110] zone-axis, (**b**) EBPlas-UV with FT pattern of an Al-nanocrystal in [110] zone-axis, (**c**) EBLes with FT pattern of γ-Al_2_O_3_ in the [101] zone-axis and (**d**) SPUT with FT pattern of γ-Al_2_O_3_ in the [103] zone-axis.

High-resolution TEM (HRTEM) images in Fig. 2 reveal further structural details of the AlO_x_-layers. EBPlas and EBPlas-UV contain crystalline regions of a few nm size embedded in the amorphous AlO_x_-matrix (nanocrystals marked by circles in Fig. 2a,b).

The crystal structure of the nanocrystals was determined by comparing the two-dimensional Fourier transformation (FT) pattern of these regions with calculated diffraction patterns. The FT patterns of all analyzed nanocrystals in EBPlas and EBPlas-UV (cf. representative examples in Fig. 2a,b) agree with calculated diffraction patterns of Al (face-centered cubic structure, space group Fm-3m, lattice parameter a = 4.06 Å^30^). An almost entirely crystalline AlO_x_-layer is found in EBLes (Fig. 2c) where electron-beam deposition from a stoichiometric Al_2_O_3_-target was used for AlO_x_-layer deposition. FT analysis (inset in Fig. 2c) shows that the AlO_x_ crystallizes in the γ-Al_2_O_3_ phase with a defect cubic spinel structure (space group Fd-3m, lattice parameter a = 7.91 Å^31^). The AlO_x_-layer of the sample SPUT (Fig. 2d) is subdivided into an amorphous lower part with an average thickness of 8.6 ± 2.1 nm and a crystalline upper part, which consists of γ-Al_2_O_3_.

**Figure 3:**
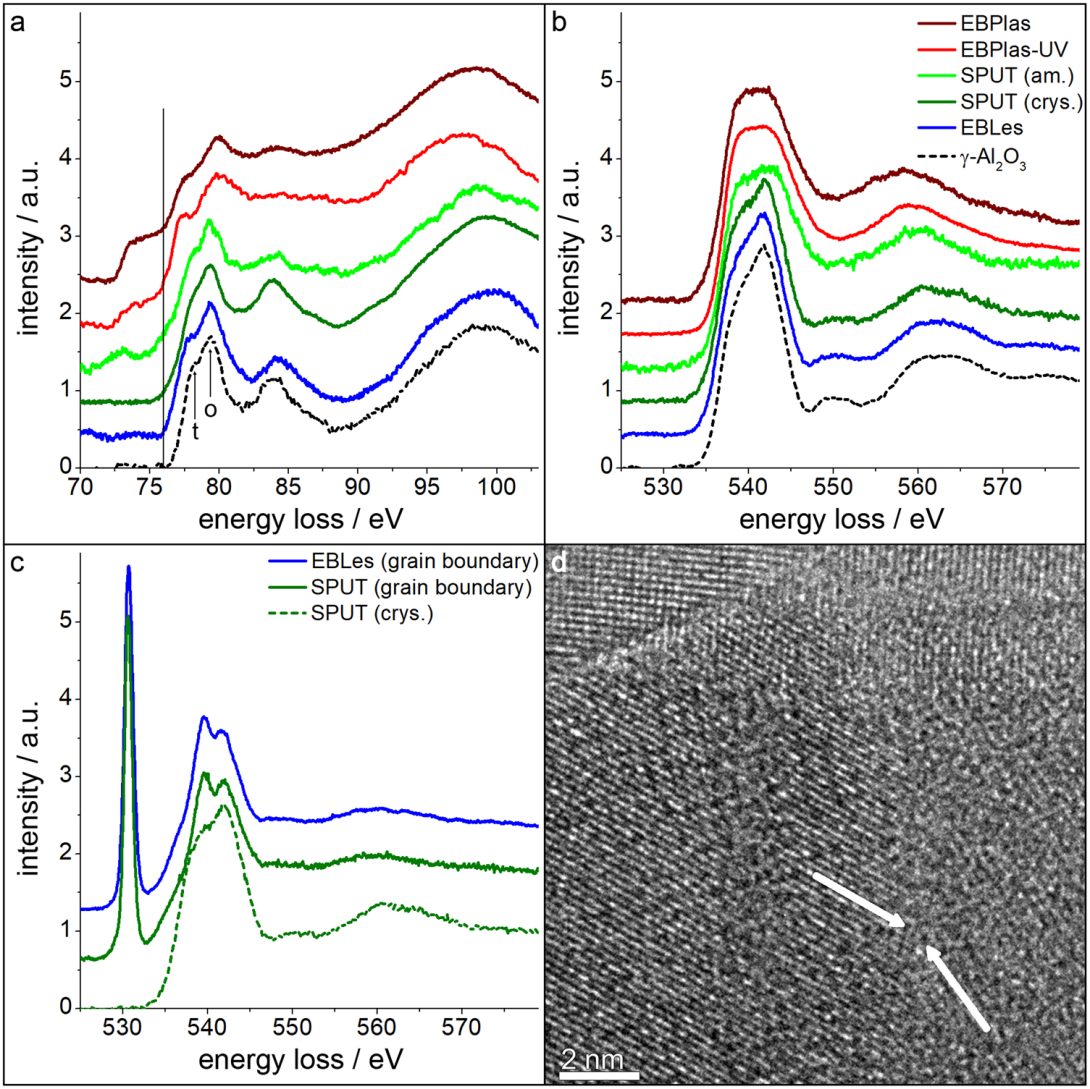
EELS spectra showing the ELNES of the Al-L_2,3_ and O-K edges in the AlO_x_-layers of all samples and a γ-Al_2_O_3_ reference specimen. Spectra of (**a**) Al-L_2,3_ and (**b**) O-K edges acquired in amorphous (EBPlas, EBPlas-UV and SPUT) and crystalline (EBLes and SPUT) regions. A spectrum of a crystalline γ-Al_2_O_3_ reference specimen is included. The edge onset of γ-Al_2_O_3_ at 76 eV is marked by a black line in (a). ‘t’ and ‘o’ correspond to tetrahedrally and octahedrally coordinated Al-atoms. (**c**) Shows the O-K edge acquired at AlO_x_-grain boundaries of EBLes and SPUT (solid lines) and within a crystalline region (dashed line) with (**d**) corresponding HRTEM image of a grain boundary region in SPUT. A boundary between two Al_2_O_3_-grains is marked by white arrows.

STEM-EELS was applied to obtain information on the local chemical composition and bonding characteristics of Al and O within the AlO_x_-layers. The energy loss near edge structure (ELNES) of the Al-L_2,3_ and O-K edges is shown in the EELS spectra (Fig. 3a–c) for all four samples. Spectra of a crystalline γ-Al_2_O_3_ reference sample obtained from a nanoparticle (nanoparticulate powder *by Carl Roth GmbH* + *Co KG, Karlsruhe, GER*) are included for comparison.

The Al-L_2,3_ edge of γ-Al_2_O_3_ starts at 76 eV (Fig. 3a, black line). The arrows mark two signals at energy losses of 77.9 eV (arrow labeled ‘t’) and 79.4 eV (arrow labeled ‘o’) which can be associated with tetrahedrally and octahedrally coordinated Al-atoms^32,33^ as expected in γ-Al_2_O_3_. The Al-L_2,3_ edge of EBLes and the crystalline AlO_x_-region of SPUT (blue and green lines in Fig. 3a) agree well with the ELNES of γ-Al_2_O_3_. The ELNES of amorphous AlO_x_ in EBPlas and EBPlas-UV (brown and red line in Fig. 3a) looks distinctly different. The onset is shifted to 72.5 eV which indicates that metallic Al is also present in the analyzed region^33^.

The pronounced maximum at 84 eV in γ-Al_2_O_3_ is not present anymore in amorphous AlO_x_ due to the lack of a medium-range ordered structure^34^. The first peak after the edge onset contains signatures of tetrahedrally and octahedrally coordinated Al-atoms but with slightly shifted energies. These changes can be attributed to distorted bonds (variations of bond lengths and angles) in the amorphous material. Moreover, Zheng *et al*.^26^ have shown that 2-, 3- and 5-fold coordinated Al-atoms are also present in amorphous AlO_x_ which are expected to influence the Al-L_2,3_ ELNES. Figure 3b presents spectra of the O-K edge of all samples including the γ-Al_2_O_3_ reference sample. The ELNES features of EBLes and the crystalline AlO_x_-region of SPUT agree well with the O-K edge of γ-Al_2_O_3_. It is characterized by an intense peak at the edge onset between 533 and 534 eV and two maxima at energy losses of 550 and 563 eV. The O-K edge in the amorphous AlO_x_-layer in EBPlas and EBPlas-UV is different and shows a broadened first peak followed by only one maximum at an energy loss of 557 eV.

Figure 3c shows again spectra of the O-K edge acquired in EBLes and SPUT. It contains two spectra that were taken with the electron beam positioned at boundaries between crystalline Al_2_O_3_-grains as indicated by the white arrows in the HRTEM image of SPUT (cf. Fig. 3d). A dramatic change of the ELNES of the O-K edge is observed at such positions (spectra plotted with solid lines in Fig. 3c) compared to the O-K edge inside crystalline γ-Al_2_O_3_ grains (spectrum with dashed line in Fig. 3c). A sharp first peak at 530.7 eV shows up at grain boundaries and a distinct peak splitting with maxima at 539.6 eV and 541.9 eV is observed. The sharp features of the O-K edge at grain boundaries indicate that molecular oxygen (O_2_) is the origin of the specific ELNES in this case. This assumption is confirmed by literature data of XAS and EELS spectra of O_2_^35,36^. In X-ray absorption spectroscopy of O_2_, the same features are observed, which correspond to transitions from the occupied 1 s to unoccupied π* (sharp line at 530.5 eV) and σ* states (539.5 and 542 eV)^37^. We like to emphasize at this point that O_2_ is inherently present at grain boundaries and is not an effect of electron-beam damage because the sharp spectral features do not develop in the course of electron-beam illumination and do not change even during extended observation in the electron microscope.

The composition of the AlO_x_-layers was quantitatively analyzed by evaluation of the intensities of the Al-L_2,3_ and O-K edges (cf. Table 1). A high O-deficiency with a chemical composition of AlO_0.5_ is found in EBPlas. The O-content raises to AlO_1.1_ in EBPlas-UV due to UV-enhanced oxidation. Small amorphous regions in EBLes consist of AlO_1.3_, which is similar to AlO_1.2_ in the lower amorphous part of the AlO_x_-layer in SPUT. Stoichiometric AlO_1.5_ is formed in the crystalline regions of EBLes and SPUT. O-excess is present at boundaries between crystalline grains of SPUT (AlO_1.8_) and EBLes (AlO_1.7_) which is consistent with the observation of molecular O_2_ in EELS spectra (Fig. 3c). Overall, amorphous AlO_x_ exhibits an O-deficiency, which depends on deposition technique and fabrication conditions. Crystalline regions consist of pure Al or stoichiometric γ-Al_2_O_3_. Grain boundaries in crystalline γ-Al_2_O_3_ show an O-excess due to the presence of molecular O_2_.

**Table 1:** Chemical composition in different regions of the AlO_x_-layers in the investigated samples.

sample	region	chemical composition AlO_x_
EBPlas	amorphous	0.48 ± 0.04
	crystalline	Al
EBPlas-UV	amorphous	1.10 ± 0.06
	crystalline	Al
EBLes	amorphous	1.31 ± 0.03
	crystalline	1.50 ± 0.04
	grain boundaries	1.73 ± 0.10
SPUT	amorphous	1.18 ± 0.05
	crystalline	1.49 ± 0.03
	grain boundaries	1.82 ± 0.10

To investigate the impact of these different microstructures on the low-energy excitations in the material, dielectric measurements at low temperatures were performed. Numerous previous studies of amorphous solids at low temperatures^11,12,38^ have demonstrated that capacitance variations allow to derive the dielectric TLS contribution to ${\epsilon }$, which is given by

1 $${\epsilon }={{\epsilon }}_{0}\cdot {{\epsilon }}_{r}={{\epsilon }}_{0}(1+{\chi }_{A{l}_{2}{O}_{3}}+{\chi }_{TLS})$$

where ${\chi }_{A{l}_{2}{O}_{3}}$ describes the temperature-independent susceptibility for the Al_2_O_3_-background. The temperature-dependent susceptibility ${\chi }_{TLS}(T)$, given by^14^

2 $${\chi }_{TLS}(T)=\kappa \cdot \,\mathrm{log}(\frac{{T}_{0}}{T})$$

is attributed to TLS at low temperatures. The prefactor *κ* ∝ *N*·*p*^2^ combines the TLS density of states per volume and energy $N$ and the squared average TLS dipole moment *p*^2 ^^15^. The TLS contribution to the dielectric permittivity ${\epsilon }$ of the AlO_x_-layer can be derived from capacitance measurements at low temperature for an applied electric field of constant frequency and amplitude. The capacitance variation ${\rm{\Delta }}C=C(T)-{C}_{{T}_{0}}$ is measured with respect to reference capacity ${C}_{{T}_{0}}\,$at an arbitrary temperature ${T}_{0}$ and leads to

3 $${\rm{\Delta }}C/{C}_{{T}_{0}}={\rm{\Delta }}{\epsilon }/{{\epsilon }}_{{T}_{0}}={{\rm{\Delta }}{\rm{\chi }}}_{{\rm{TLS}}}/{{\epsilon }}_{{T}_{0}}$$

as ${\chi }_{A{l}_{2}{O}_{3}}$ is expected to be constant and therefore ${\rm{\Delta }}{\chi }_{A{l}_{2}{O}_{3}}=0$.

**Figure 4:**
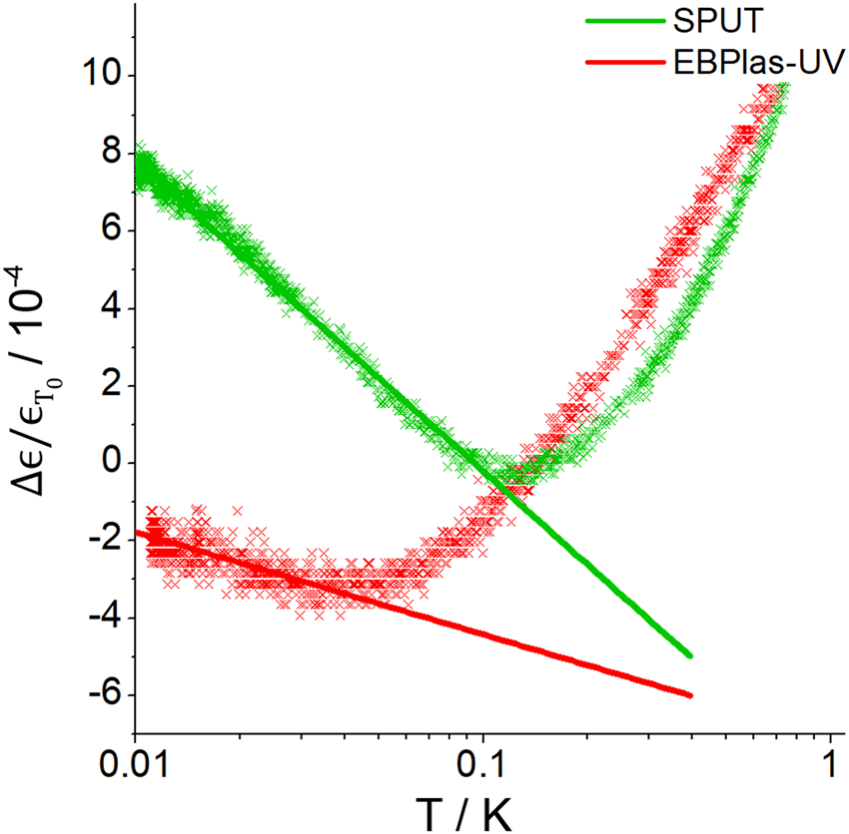
Low-temperature dielectric capacitance measurements of EBPlas-UV and SPUT. ${\Delta }\epsilon /{\epsilon }_{{{T}}_{0}}$ is plotted as a function of the logarithm of the temperature. The solid lines are linearly fitted to the data and represent the slope *κ* of ${\Delta }\epsilon /{\epsilon }_{{{\boldsymbol{T}}}_{0}}\,$ at low temperatures.

Figure 4 shows the variation of the dielectric permittivity ${\rm{\Delta }}{\epsilon }$ as a function of the logarithm of the temperature. ${\rm{\Delta }}{\epsilon }$ is normalized with ${{\epsilon }}_{{T}_{0}}$ where ${T}_{0}$ was chosen to be 200 mK for SPUT and EBPlas-UV. The capacitance measurements were performed at 1 kHz and an applied voltage of 1 mV. The dielectric response of the disordered AlO_x_-layers shows signatures similar to glasses which allows to extract the underlying TLS properties and their dependence on the fabrication method as pointed out in Eqs (1–3). Starting from the lowest temperature the dielectric permittivity decreases with rising temperature, because TLS with energy splitting $E$ are thermally excited when ${k}_{B}T$ becomes larger than $E$. Therefore TLS cannot be polarized by the applied electric field. Due to the broad distribution of TLS energies, this decrease depends on the logarithm of the temperature and is proportional to $\kappa$ according to Eq. (2). A minimum of ${\rm{\Delta }}{\epsilon }$ appears when the relaxation rate of the dominating TLS with $E\approx {k}_{B}T$ becomes equal to the oscillating frequency of the applied electric field. At even higher temperatures the energy relaxation rate of TLS exceeds the frequency of the external field and leads to an increase of the permittivity $\Delta {\epsilon }$. At temperatures ${T}_{min}$, where the permittivity minimum occurs, the TLS relaxation is dominated by one-phonon processes. ${T}_{min}$ gives therefore a measure of the coupling strength of TLS to phonons – the smaller ${T}_{min}$, the stronger the coupling.

The temperature dependences of the dielectric permittivity of SPUT and EBPlas-UV are remarkably different (cf. Fig. 4) in two aspects. EBPlas-UV shows a much weaker decrease of the dielectric permittivity than SPUT indicating that $\kappa$ is reduced in EBPlas-UV. In addition, the TLS relaxation rate for EBPlas-UV exceeds the frequency of the applied ac-electric field of 1 kHz at 50 mK – a behavior that is known from many other disordered solids. However, the onset of relaxation in SPUT at 150 mK indicates an unusual weak coupling to phonons. This difference in coupling strength to phonons between SPUT and EBPlas-UV can be most likely attributed to different microscopic origins of the relevant TLS.

For EBPlas the TLS contributions to the dielectric permittivity could not be determined due to a high dc-loss, which obscures small changes of the capacitance caused by dielectric TLS contributions to ${\epsilon }$. This could be the result of an increased conductivity due to the low O-content (AlO_0.5_) in the AlO_x_-layer. In addition, the high volume fraction of crystalline Al-inclusions could lead to the formation of conducting channels through the AlO_x_-layer and thus to a high dc-conductivity during low-temperature measurements.

The dc-loss can also be caused by problems with the epoxy used for contacting the capacitor. Although the used epoxy is specified for this purpose, a color chance of the upper Al-layer was visible, which indicates a chemical reaction between epoxy and upper Al-layer.

For EBLes the lithography process failed and sample preparation for capacitance measurements was not possible. This can be attributed to strong thickness variations of the Al_2_O_3_-layer, which lead to problems with wet-etching of the upper Al-layer and may induce electrical shortcuts by accidental complete removal of thin sections of the Al_2_O_3_-layer.

## Discussion

Analytical TEM shows that the nanostructural and nanochemical properties of AlO_x_-layers in Al/AlO_x_/Al-layer systems vary considerably depending on the deposition system and conditions. In the following we will first discuss the properties of the lower Al-layer and their effect on the AlO_x_-layers. We then focus on the properties of the AlO_x_-layers and finally discuss the correlation of the measured dielectric TLS contribution $\kappa$ of samples EBPlas-UV and SPUT with their nanoscale structural and chemical properties.

### Properties of the lower Al-layer

Figure 1a–d demonstrate that the morphology of the AlO_x_-layers is strongly influenced by the lower Al-layer and motivate efforts to optimize its properties. AlO_x_-thickness variations strongly affect the local tunnel current, which scales as ${e}^{-t/{\rm{\lambda }}}$ with the local barrier thickness *t* and the attenuation length λ. Zheng *et al*.^24^ have already shown that less than 10% of the total barrier area is active in the tunneling process due to thickness fluctuations in the tunnel barriers of JJs studied in their work.

Pronounced AlO_x_-thickness variations occur at grain boundaries of the underlying Al-layer (cf. regions indicated by arrows Fig. 1a) or on bottom Al-layers with strongly corrugated surface topography (cf. Fig. 1c). AlO_x_-layer sections with homogeneous thickness are observed on top of single Al-grains with planar surface. There are two possible origins for the thickness increase of AlO_x_ at Al-grain boundaries. One effect is grain boundary grooving due to the establishment of a local mechanical equilibrium between Al-surface tension and grain boundary energy. The effect was originally studied by Mullins^39^ and recently discussed with respect to AlO_x_-layers in JJs by Nik *et al*.^40^. Another possibility can be enhanced oxygen diffusion along grain boundaries^41^. Irrespective of the mechanism, large lateral grain sizes with homogeneous thickness are desirable to avoid fluctuations of the AlO_x_-thickness and tunnel current caused by it. Nik *et al*.^40^ observed a log-normal grain size distribution and found the average lateral grain sizes to be approximately by two to three times larger than the Al-layer thickness. These characteristics are typical for normal grain growth^42^, which is also observed in our samples EBPlas, EBPlas-UV and SPUT where a log-normal grain size distribution and lateral average grain sizes of twice the Al-film thickness were found.

The microstructure of the Al-layer in EBLes clearly deviates from the behavior of the other samples because the average grain size of 54 nm corresponds to only 50% of the film thickness and the surface is strongly corrugated. This is on first sight surprising because EBLes was also fabricated by electron-beam deposition as EBPlas and EBPlas-UV (although in a different system) with similar nominal deposition rates (0.2 nm/s for EBPlas and EBPlas-UV vs. 0.13 nm/s for EBLes) without intentional cooling or heating of the substrate. We attribute the observed differences to the effect of different O_2_-partial pressures during Al-deposition as previously reported for electron-beam deposited Al-films by Verkerk *et al*.^43^. Al-oxidation can take place at higher O_2_-partial pressures during Al-deposition, which then initiates the formation of secondary Al-grains on the oxidized Al-surface. Nucleation of new Al-grains within the Al-film is clearly recognized in Fig. 1c and contributes to the reduction of the average grain size. Oxidation may indeed have occurred during Al-deposition in the Lesker electron-beam deposition system where the (overall) pressure of 1.5 10^−6^ mbar was one order of magnitude higher than in the Plassys system (1.5 10^−7^ mbar). Oxidation may have been also favored by the smaller deposition rate of 0.13 nm/s in the Lesker system compared to 0.2 nm/s in the Plassys system. Another origin of the reduced average grain size may be found in the substrate properties. In addition to the native SiO_x_-layer, a 10–13 nm thick carbon contamination layer was present between bottom Al-layer and substrate for EBLes, despite identical substrate pretreatment. The larger substrate roughness provides a higher density of grain nucleation sites and contributes to the observed small average grain size.

### Properties of the Al-oxide layers

The composition of the AlO_x_-layer tunnel barrier in JJs could be a decisive property for the TLS density as suggested by theoretical studies^12^. The measured compositions (cf. Table 1) reveal substantial O-deficiencies depending on the oxidation procedure. All amorphous AlO_x-_layers are sub-stoichiometric in contrast to γ-Al_2_O_3_ in EBLes and SPUT, which contain the expected O-concentration. Dynamic oxidation with O_2_ with 10 sccm for 12.5 min for EBPlas yields strongly O-deficient amorphous AlO_0.5_. The O-concentration increases considerably to AlO_1.1_ if UV-illumination is used (EBPlas-UV) in combination with a slightly increased O_2_-flux of 12.7 sscm. The interaction of UV-photons and O_2_ enhances the dissociation rate of O_2_ at the surface and additionally creates energetically activated O-ions which help to reduce the activation barrier for chemisorption^44^. The resulting negatively charged O-ions diffuse into the AlO_x_-layer and bind to positively charged Al-ions. This effect can be further enhanced by low-energy electron-bombardment of the surface to drive the charged O-ions deeper into the AlO_x_-layer^20^.

Monocrystalline Al-inclusions with a few nm size are embedded in the amorphous AlO_x_-layer of EBPlas and EBPlas-UV (cf. Fig. 2a,b). They are only present in samples fabricated in the Plassys system where repeated Al-deposition/dynamic-oxidation (cf. Methods) were applied to obtain AlO_x_-layers with sufficient thickness and high resistance. TEM studies of samples obtained with a single dynamic oxidation process (not presented here) show that the AlO_x_-layer has a maximum thickness of about 3 nm. To obtain thicker AlO_x_-layers, approximately 1 nm Al is deposited after the first oxidation step and dynamically oxidized subsequently. This process step is repeated up to ten times resulting in a ~20 nm thick AlO_x_-layer. We assume that a 1 nm Al-deposition does not lead to a homogenous Al-coverage but to the formation of Al-islands, which are not fully oxidized in the subsequent oxidation process leading to embedded Al-inclusions.

The amorphous lower part of SPUT with a composition of AlO_1.2_ contains a rather high O-concentration. The enhancement of O-concentration compared to dynamic oxidation is attributed to the plasma-assisted deposition process where several factors contribute to the high O-content. First, the Ar/O-plasma generates dissociated and more reactive O-ions at the AlO_x_-surface. Second, a non-negligible concentration of the sputtered Al-atoms could be already oxidized in the gas phase and, third, the surface of the Al-sputter target may be partially oxidized during the sputter process.

An interesting observation is the transition from amorphous AlO_1.2_ to crystalline γ-Al_2_O_3_ (cf. Fig. 2c) on a substrate that was not intentionally heated during the deposition process. A clue to understand this phenomenon is provided by Jeurgens *et al*.^45^. They calculated the thermodynamic stability of amorphous and crystalline Al_2_O_3_ on Al-surfaces with different crystallographic orientation. By considering the total Gibbs free energy of amorphous and crystalline Al_2_O_3_-films they found amorphous Al_2_O_3_ to be more stable than γ-Al_2_O_3_ up to a critical thickness that depends on the crystallographic orientation of the Al-surface and the temperature. Typical calculated values for the critical thickness at room temperature are 2 nm for Al(100) and 4 nm for Al(110) which are in reasonable agreement with the measured thickness of 8.6 ± 2.1 nm taking into account that the polycrystalline Al-film provides a variety of different, not necessarily low-index surfaces.

Interestingly, EBLes contains an almost completely nanocrystalline γ-Al_2_O_3_ layer with only small amorphous regions which was obtained without intentional substrate heating. We attribute its formation to the comparatively high pressure of 10^−5^ mbar during oxidation in Lesker system (higher base pressure and evaporation from Al_2_O_3_-pellets) and the very low evaporation rate of 0.03–0.04 nm/s. This observation underlines that high temperatures are not required for γ-Al_2_O_3_ formation. An extremely slow growth rate in an atmosphere with a sufficient O_2_-concentration facilitates uptake of a rather large concentration of oxygen that is favorable for the formation of crystalline γ-Al_2_O_3_. Although the formation of a crystalline Al_2_O_3_-layer is on first sight desirable, the discussion in the next section will show that O_2_ embedded in grain boundaries has a detrimental effect on $\kappa$ as shown by the different dielectric behavior of EBPlas-UV and SPUT (cf. Fig. 4).

### Correlation of dielectric TLS contribution and Al-oxide nanostructure

In the following we discuss the correlation between nanoscale structural and chemical properties and dielectric permittivity for EBPlas-UV and SPUT. With respect to the possible origin of TLS, DuBois *et al*.^12^ suggested delocalized O-atoms in amorphous AlO_x_ as possible sources of TLS where O-atoms in an Al-O-Al chain can occupy different positions and induce this way a TLS and charge dipole. Another indication that oxygen is connected with TLS was provided by the study of Tan *et al*.^20^ who detected weakly bound O-atoms by X-ray photoelectron studies and suggested that these O-atoms could be metastable defects and contribute to $1/f$ noise in JJs. These previous studies suggest to focus on the O-content and O-bonding to understand the strongly different dielectric permittivity of SPUT and EBPlas-UV.

SPUT contains a complex AlO_x_-layer where the upper part consists of nanocrystalline γ-Al_2_O_3_ with a considerable content of O_2_ located at grain boundaries (cf. Table 1 and Fig. 3c). The lower part with an average thickness of 8.6 nm (about 40% of the overall thickness) is amorphous with a composition of AlO_1.2_. The AlO_x_-layer in EBPlas-UV is amorphous with some embedded nanocrystalline Al-inclusions. The O-concentrations of SPUT (AlO_1.2_) and EBPlas-UV (AlO_1.1_) agree within the error limits and suggest comparable amorphous AlO_x_-properties. Assuming that the O-content is correlated with the defect concentration that determines the TLS density, we expect similar values $\kappa$ values for both samples. However, the dielectric measurements (Fig. 4) show that $\kappa$ is much larger in SPUT than in EBLes-UV. This suggests that the crystalline part of the AlO_x_-layer in SPUT is responsible for the higher value $\kappa$. Thus, it either contains a higher TLS density of states in the crystalline layer or the individual TLS must have a larger electric dipole moment. While crystalline Al_2_O_3_ has shown beneficial effects if present as epitaxial layer (80% reduced TLS density^46^ and reduced TLS coupling strength^47^), the nanocrystalline γ-Al_2_O_3_ contains a high density of grain boundaries with embedded O_2_. We tentatively interpret the high $\kappa$ to be induced by loosely bound oxygen that forms atomic tunneling systems. As the number of O-atoms at grain boundaries should be much smaller than the number of defects in an amorphous layer, it is likely that TLS located at γ-Al_2_O_3_ grain boundaries have a large dipole moment due to the high electronegativity of the oxygen atoms. The observation of the unusual high temperature of 150 mK for the onset of relaxation processes in the dielectric measurements supports this interpretation, because such loosely bound O-atoms may couple rather weakly to lattice vibrations (cf. Fig. 4). This interpretation is in line with recent work by Kumar *et al*.^48^ who found O_2_ on the surface of Al/AlO_x_/Al-based qubits to be the dominant source of $1/f$ noise in their structures.

## Conclusions

In this work, different deposition techniques were applied to fabricate Al/AlO_x_/Al-structures with different properties. The structures were analyzed by transmission electron microscopy combined with electron energy loss spectroscopy to reveal structure and chemistry on the nanoscale. Low-temperature capacitance measurements were demonstrated to be sensitive towards the contribution of TLS to the dielectric susceptibility in AlO_x_. The combination of these techniques was applied for the first time and is promising to give hints on the structural and/or chemical origin of TLS as a prerequisite to systematically reduce the TLS density. Specifically, the correlation of the TLS contribution $\kappa$ to the dielectric susceptibility and structural details reveals that O_2_ in grain boundaries of nanocrystalline γ-Al_2_O_3_ is one possible origin of TLS with a high dipole moment and thus can be a cause for $1/f$ noise in superconducting quantum circuits.

The correlation of nanostructure and nanochemistry with fabrication conditions demonstrate the strong impact of the fabrication conditions on the properties of Al/AlO_x_/Al-layer systems and allow conclusions with respect to optimization of Al/AlO_x_/Al-structures for JJs as summarized in the following:

- The morphology of the AlO_x_-layer is predominantly determined by the structure and morphology of the bottom Al-layer and even the surface morphology of the underlying substrate plays a role. Grain boundaries intersecting the lower AlO_x_/Al-interface lead to thickness variations of the AlO_x_-layer. AlO_x_-layers with homogeneous thickness can be expected from a flat and, ideally, epitaxial bottom Al-electrode layer without grain boundaries.
- The O-content in amorphous AlO_x_ varies strongly depending on the deposition conditions. The highest O-contents were obtained by UV-assisted dynamic oxidation of Al and sputter-deposition in an Ar/O-plasma. Since the TLS density is likely to be correlated with the defect density, the O-content in amorphous AlO_x_ should be as close as possible to AlO_1.5_ to minimize the concentration of low-coordinated atoms.
- Nanocrystalline γ-Al_2_O_3_ was obtained without intentionally heating the substrate, either by electron-beam evaporation from Al_2_O_3_ pellets or by a strain-induced transition from amorphous to crystalline Al-oxide. However, nanocrystalline Al_2_O_3_ is not well suited for JJ tunnel barriers in superconducting quantum circuits because oxygen molecules are present in grain boundaries which are possible sources of TLS with a large dipole moment. Only single crystalline Al_2_O_3_ tunnel barriers are beneficial to reduce the TLS density.

## Methods

### Fabrication of Al/AlO_x_/Al-layer systems

All Al/AlO_x_/Al-layer systems were fabricated on single crystalline Si(001)-substrates. The substrates were cleaned with N-ethyl-2-pyrrolidon (NEP), isopropyl alcohol and water to remove the protective resist. All substrates are covered with a 3–4 nm thick native SiO_x_-layer which forms even after short exposure to air.

The Al/AlO_x_/Al-structures were fabricated in three different deposition systems. A *MEB 550S* (*PLASSYS Bestek, Marolles-en-Hurepoix, FR*) electron-beam evaporation system was used for the samples denoted by EBPlas and EBPlas-UV with a base pressure of 10^-7^ mbar. The whole fabrication procedure was carried out without cooling or heating the substrate holder. After the deposition of the bottom Al-layer with a deposition rate of 0.2 nm/s (measured by a piezoelectric sensor) at a deposition pressure of 10^−7^ mbar, the surface was dynamically oxidized by supplying O_2_ with 10 sccm for 12.5 min. The deposition rate for the top Al-layer was also 0.2 nm/s. The second sample, EBPlas-UV, was fabricated with a slightly increased O_2_-flux of 12.7 sccm with the same oxidation time of 12.5 min, combined with additional UV-illumination. To remove contamination caused by the bakeout of the UV-lamp, the Si-substrate was plasma-cleaned for 6 min prior to the deposition of the bottom Al-layer. However, the plasma cannot remove the 3–4 nm of SiO_x_-layer covering the substrate surface. AlO_x_-layers with thicknesses of ~20 nm for dielectric measurements were obtained by repeated deposition of 1 nm Al and oxidation for up to 10 times.

For comparison, an Al/AlO_x_/Al-layer system, denoted by EBLes, was fabricated in a different electron-beam evaporation system (*PVD 75, Kurt J. Lesker Company, Hastings, UK*). The Al-layers were deposited with a low rate of 0.13 nm/s at a pressure between 1.0 and 1.5·10^−6^ mbar using a BN-TiB_2_ crucible with Al-pellets. The AlO_x_-layer was also deposited by electron-beam evaporation using a second crucible with Al_2_O_3_-pellets and a deposition rate of 0.03–0.04 nm/s at a pressure of 10^−5^ mbar.

The sample SPUT was fabricated in a home-built sputter deposition system. The substrate was plasma-cleaned with an Ar-flux of 14 sccm at 20 W for 2 min. The deposition of the bottom and top Al-layers took place by Ar-sputtering with an Ar-flux of 19 sccm and a rate of 0.6 nm/s (top layer) and 0.5 nm/s (bottom layer) at 10^−3^ mbar. The Al-target was also used for sputter deposition of the AlO_x_-layer by using an Ar/O-plasma (9:1 mixture) at 10 sccm in addition to an increased Ar-flux of 33 sccm at a pressure of 1.4·10^−2^ mbar resulting in a deposition rate of 0.45 nm/s.

### Transmission Electron Microscopy and Electron Energy Loss Spectroscopy

Transmission electron microscopy (TEM) was performed with a FEI Titan^3^ 80–300 microscope operated at 300 kV, which is equipped with an aberration corrector in the imaging lens systems and with a Gatan imaging filter Tridiem HR 865 for electron energy loss spectroscopy (EELS). TEM cross-section specimens were prepared by standard mechanical preparation techniques^49^ and Ar^+^-ion milling with ion energies between 0.5 and 3 keV as final preparation step. Surface oxidation of TEM cross-section samples during transfer from Ar^+^-ion mill to the microscope is negligible because EELS spectra of Al-layers do not show an O-K signal above the noise level. Structure analyses of nanoscale crystalline regions in the AlO_x_-layers were performed by comparing two-dimensional Fourier transformation (FT) patterns of HRTEM images with simulated diffraction patterns using the *jems* software^50^.

EELS in the scanning transmission electron microscopy (STEM) mode was performed with a convergence angle of 16.7 mrad of the electron probe and a spectrometer acceptance angle of 20.3 mrad. Spectra in Fig. 3 were acquired with a dispersion of 0.02 eV/channel for the Al-L_2,3_ edge and 0.05 eV/channel for the O-K edge. For quantitative composition analysis, the Al-L_2,3_ and O-K should be acquired within a single spectrum with high energy resolution. This is not possible because a dispersion of 0.5 eV/channel is necessary to resolve both edges and the count rate of the O-K edge is lower by two orders of magnitude compared to the Al-L_2,3_ edge. Therefore, Al-L_2,3_ and O-K edges were acquired consecutively. To optimize spectrum acquisition for quantitative composition measurement, a self-written script was used which acquires ten to fifty Al-L_2,3_ and O-K spectra alternatingly with a dispersion of 0.05 eV/channel with acquisition times of 0.1 s for the Al-L_2,3_ edge and up to 10 s for the O-K edge combined with binned-gain averaging^51^. The local stoichiometry of the AlO_x_-layers was quantified on the basis of the k-factor method^52^ where γ- and α-Al_2_O_3_ with a known composition were used as a reference material. The intensities were measured by signal integration over energy-loss windows with widths between 30 eV and 60 eV taking the different acquisition times for the Al-L_2,3_ edge and O-K edge into account. The variation of the energy-loss integration window width leads to variations of the evaluated composition between 3–5%. Fluctuations of the electron-beam current or microscope alignments contribute to the error, which can also be minimized by alternating acquisition of the two edges. The total error of the chemical composition is estimated to be less than 10%. We note that the intensity of the sharp peak at 530.7 eV in O-K spectra (Fig. 3c) was not included in the composition quantification because this peak is considered to be an energy loss near edge structure (ELNES) feature that would artificially increase the O-content. Composition data given in Table 1 are based on averaging five to ten measurements at different positions in the center of the AlO_x_-films.

### Dielectric Measurements

The Al/AlO_x_/Al-layer systems of EBPlas, EBPlas-UV and EBLes were deposited to cover the full chip area. Two plate capacitors in series were fabricated for capacity measurements. For this purpose, two areas of each ~1 mm² were covered with photoresist Microposit S-1805 (*Dow Chemical Company, Midland, USA*) and wet-etched to remove only the uncovered upper Al-layer using Microposit M-319 (*Dow Chemical Company, Midland, USA*). After removal of the upper Al-layer, the etching process is stopped by a dip in distilled water. To determine the etching-time, a test chip for each sample was used where the whole layer-system was removed, because the difference between AlO_x_-layer and Al-layer cannot be seen during the etching but the Si-substrate is clearly visible. Only 50% of total etching time was then used for etching our capacitors. As the AlO_x_ etching-rate is lower by about one order of magnitude, after half of the total etching time, the upper Al-layer is completely removed while the lower Al-layer is still intact. After removal of the photoresist the two remaining areas of the upper Al-layer were contacted by attaching copper wires with silver epoxy EPO-TEK H21 (*Epoxy Technology Inc., Billerica, USA*).

The samples were mounted in a dilution cryostat and capacitance and loss were measured using an Andeen-Hagerling 2500 A (*Andeen-Hagerling Inc., Cleveland, USA*) precision capacitance bridge at 1 kHz. For temperature-dependent capacitance measurements, the cryostat is slowly cooled from 1 K to 10 mK within 10–12 h to keep thermal equilibrium with the sample. During this process temperature, capacitance and dielectric loss were measured every 2 min with an acquisition time of 20 s. The temperature was determined by several calibrated temperature-dependent resistors. Using the plate capacitor geometry, the normalized variation of the permittivity can be derived from the capacity according to Eqs (1–3).
